# In Vitro 3D *Staphylococcus aureus* Abscess Communities Induce Bone Marrow Cells to Expand into Myeloid-Derived Suppressor Cells

**DOI:** 10.3390/pathogens10111446

**Published:** 2021-11-06

**Authors:** Marloes I. Hofstee, Anja Heider, Sonja Häckel, Caroline Constant, Martijn Riool, R. Geoff Richards, T. Fintan Moriarty, Sebastian A. J. Zaat

**Affiliations:** 1 AO Research Institute Davos, 7270 Davos, Switzerland; marloes.hofstee@aofoundation.org (M.I.H.); caroline.constant@aofoundation.org (C.C.); geoff.richards@aofoundation.org (R.G.R.); 2 Department of Medical Microbiology and Infection Prevention, Amsterdam UMC, Amsterdam Institute for Infection and Immunity, University of Amsterdam, 1105 AZ Amsterdam, The Netherlands; m.riool@amsterdamumc.nl (M.R.); s.a.zaat@amsterdamumc.nl (S.A.J.Z.); 3 Swiss Institute of Allergy and Asthma Research (SIAF), University of Zurich, 7265 Davos, Switzerland; anja.heider@siaf.uzh.ch; 4 Department of Orthopaedic Surgery and Traumatology, Inselspital Bern, 3010 Bern, Switzerland; sonja.haeckel@insel.ch

**Keywords:** *Staphylococcus aureus*, staphylococcal abscess community, myeloid-derived suppressor cell, 3D in vitro model, bone infection, host-pathogen interaction

## Abstract

*Staphylococcus aureus* is the main causative pathogen of subcutaneous, bone, and implant-related infections, forming structures known as staphylococcal abscess communities (SACs) within tissues that also contain immunosuppressive myeloid-derived suppressor cells (MDSCs). Although both SACs and MDSCs are present in chronic *S. aureus* infections, it remains unknown whether SACs directly trigger MDSC expansion. To investigate this, a previously developed 3D in vitro SAC model was co-cultured with murine and human bone marrow cells. Subsequently, it was shown that SAC-exposed human CD11b^low/−^ myeloid cells or SAC-exposed murine CD11b^+^ Gr-1^+^ cells were immunosuppressive mainly by reducing absolute CD4^+^ and CD8α^+^ T cell numbers, as shown in T cell proliferation assays and with flow cytometry. Monocytic MDSCs from mice with an *S. aureus* bone infection also strongly reduced CD4^+^ and CD8α^+^ T cell numbers. Using protein biomarker analysis and an immunoassay, we detected in SAC–bone marrow co-cultures high levels of GM-CSF, IL-6, VEGF, IL-1β, TNFα, IL-10, and TGF-β. Furthermore, SAC-exposed neutrophils expressed Arg-1 and SAC-exposed monocytes expressed Arg-1 and iNOS, as shown via immunofluorescent stains. Overall, this study showed that SACs cause MDSC expansion from bone marrow cells and identified possible mediators to target as an additional strategy for treating chronic *S. aureus* infections.

## 1. Introduction

*Staphylococcus aureus* is an opportunistic pathogen that can cause a range of infections, including subcutaneous, bone, and implant-related infections. A key common feature amongst these infections is biofilm and staphylococcal abscess communities (SACs) [1,2,3,4]. The outer margin of a typical abscess comprises collagen and fibrinogen, which enclose a minority of monocytes and M2 macrophages and many neutrophils, with the fibrin-encapsulated SAC at the center [3].

Myeloid-derived suppressor cells (MDSCs) are immature myeloid cells, either monocytic (M-MDSCs; CD11b^+^, Ly6C^high^, Ly6G^−^) or granulocytic (G-MDSCs; CD11b^+^, Ly6C^low^, Ly6G^+^), which suppress other immune cells, such as T cells [5,6,7]. One way MDSCs suppress T cell proliferation is by depleting L-arginine from their surrounding milieu through upregulation of the enzymes arginase-1 (Arg-1) and inducible nitric oxide synthase (iNOS), which metabolize L-arginine, a metabolite important for the T-cell receptor (TCR) zeta chain and for T cell activation [7,8,9]. MDSC expansion has been observed in patients with cancer, inflammation, autoimmune diseases, and chronic infections, and has been linked to increased myelopoiesis after a decrease in myeloid cell numbers [10]. The growth factors granulocyte macrophage colony-stimulating factor (GM-CSF), granulocyte-colony-stimulating factor (G-CSF), macrophage-colony-stimulating factor (M-CSF), and vascular endothelial growth factor (VEGF), as well as the cytokine interleukin (IL)-6, are the main drivers of MDSC expansion by activation of the transcription factor STAT3 [7,11,12]. During a bacterial infection, activation of MDSCs can either occur directly via toll-like receptor (TLR) ligands, such as *S. aureus* lipoproteins, or indirectly via prolonged exposure to pro-inflammatory cytokines such as IL-1β, TNFα, or IFNγ [12].

MDSCs were originally described in cancer patients approximately 30 years ago [10]. More recently, MDSCs have been identified in chronic *S. aureus* infections varying from subcutaneous infections [13] to implant-related biofilm or soft tissue infections [14,15,16,17]. Additionally, we showed in our recent publication that MDSCs are also present in the bone marrow of mice with an *S. aureus* osteomyelitis [3]. In that study, we showed that the neutrophils and monocytes around SACs and present within abscesses were alive, had an immature appearance, and had the typical phenotypical MDSC-markers (CD11b^+^, Ly6C^high^, Ly6G^−^ or CD11b^+^, Ly6C^low^, Ly6G^+^) [3]. Moreover, immunosuppressive regulatory T cells (Tregs) [3,18] and anti-inflammatory M2 macrophages [3,19] were observed surrounding the abscesses. These cells are commonly present in the MDSC-rich tumor microenvironment and are induced by MDSCs [10,20]. These previous studies, therefore, suggest that the cells around SACs and within the SAC-surrounding abscesses might be immunosuppressive MDSCs. However, it remains to be confirmed whether the SACs itself causes MDSCs to expand from bone marrow cells.

The aim of this study, therefore, was to examine whether SACs prime bone marrow cells to develop immunosuppressive abilities and to an MDSC phenotype using functional assays. Functional assays evaluate T cell function activity (e.g., T cell proliferation) and whether cells that potentially are MDSCs modulate this [8]. To enable studies into SAC formation, growth, and host cell interactions, a 3D in vitro SAC model was previously developed in our lab [21]. These in vitro SACs are encased within a fibrin layer and are not penetrated by neutrophils, as also observed for in vivo SACs, in addition to being resistant to high doses of the antibiotics gentamicin and rifampicin [21]. In this study, we investigated the interaction of the in vitro SAC model with both murine and human bone marrow cells to determine their immunosuppressive activity and the secreted proteins.

## 2. Results

### 2.1. Murine Bone Marrow Cells Co-Cultured with S. aureus SACs

**Figure 1:**
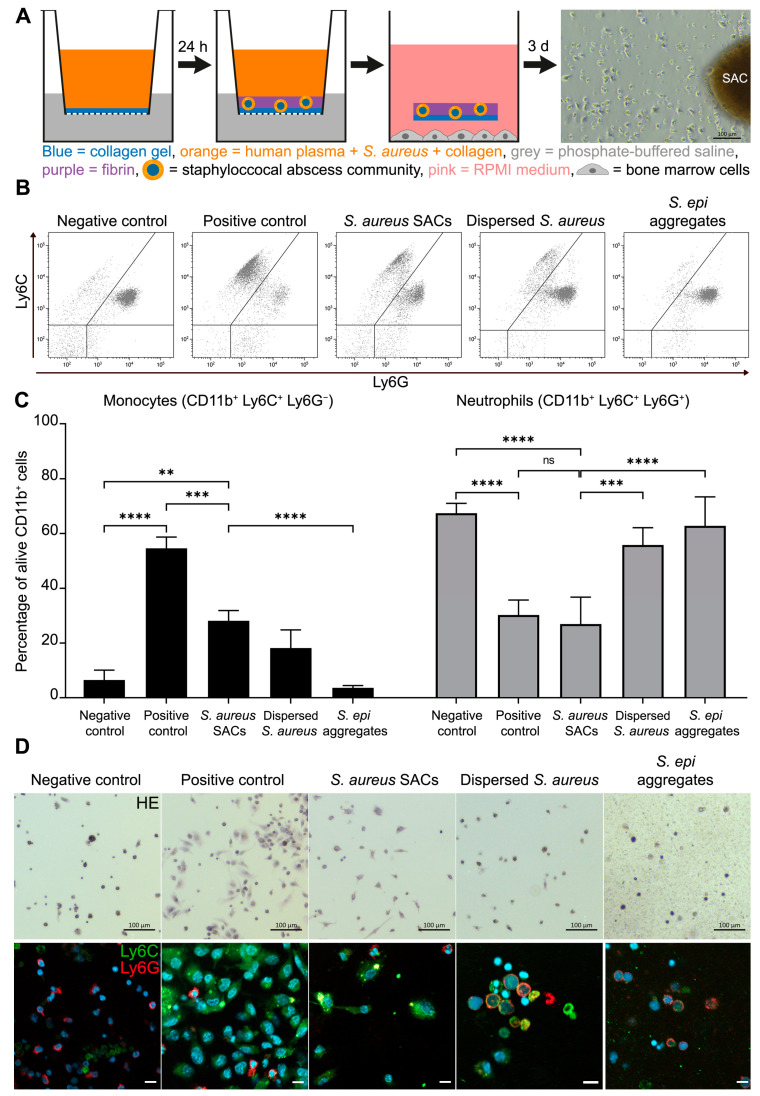
Murine bone marrow cells were co-cultured with in vitro *S. aureus* SACs. (**A**) A schematic overview of how the in vitro SACs were generated and how the bone marrow cells were co-cultured with in vitro SACs. As a comparison to this co-culture, murine bone marrow cells were kept untreated (negative control), were treated with GM-CSF and IL-6 (positive control), or were co-cultured with dispersed *S. aureus* in a collagen gel or *S. epidermidis* aggregates. The percentages of monocytes (Ly6C^+^) and neutrophils (Ly6G^+^) from alive CD11b^+^ cells were determined with flow analysis and are depicted as (**B**) flow pots or (**C**) bar graphs. Data are means (±SD) and are from three independent experiments with three replicated per test. (**D**) Negative control, positive control, *S. aureus* SAC, dispersed *S. aureus*, or *S. epidermidis* aggregate–exposed murine bone marrow cells were stained with hematoxylin and eosin (upper row) or with antibodies (lower row) for Ly6C (green; monocytes) and Ly6G (red; neutrophils) combined with DAPI as the nuclear stain (blue). Scale bar = 10 µm. Statistical test used: Tukey’s multiple comparison test; ns = non-significant, ** *p* < 0.01, *** *p* < 0.001, and **** *p* < 0.0001.

The in vitro SAC model was co-cultured with murine bone marrow cells (Figure 1A) to explore whether bacteria within a SAC can elicit bone marrow cells to adopt an MDSC phenotype. Murine bone marrow cells were either left untreated as a negative control (no MDSC expansion) or supplemented with IL-6 and GM-CSF as a positive control for MDSC expansion. Furthermore, murine bone marrow cells were co-cultured either with *S. aureus* dispersed in a collagen gel or *S. epidermidis* aggregates to determine whether the observed effects were SAC-specific. The percentages of monocytes and neutrophils from alive CD11b^+^ cells were determined with flow cytometry for the above-mentioned samples, as these might change when cells expand into MDSCs (Figure 1B,C). Compared to the negative control, the positive control and the SAC-exposed bone marrow cultures contained significantly more Ly6C^+^ monocytes (*p* < 0.0001 and *p* = 0.0028, respectively). However, the percentage of monocytes in SAC-exposed co-cultures was less than for the positive control (*p* = 0.0003) and did not differ from the numbers in the co-cultures with dispersed *S. aureus*. Co-cultures with *S. epidermidis* aggregates had a small percentage of monocytes present. Percentages of Ly6G^+^ neutrophils of the negative control were significantly higher than in the positive control and SAC-exposed cultures (*p* < 0.0001 and *p* < 0.0001, respectively), which contained similar neutrophil percentages. The percentage of neutrophils in bone marrow–SAC co-cultures was also significantly lower than in the co-cultures with dispersed *S. aureus* and with *S. epidermidis* aggregates (*p* = 0.0001 and *p* < 0.0001, respectively).

Next, the morphological appearance and a Ly6C (monocytes) and Ly6G (neutrophils) double-staining of the cells from the different conditions were examined (Figure 1D). The negative control bone marrow cells were mainly small Ly6G^+^ cells. In contrast, the positive control bone marrow cells had clusters of elongated Ly6C^+^ cells with some smaller Ly6G^+^ cells. Similar cells were observed in the bone marrow–SAC co-culture. Bone marrow cells exposed to dispersed *S. aureus* also contained a few elongated Ly6C^+^ cells but mostly round Ly6G^+^ cells were present. The bone marrow cells co-cultured with *S. epidermidis* aggregates were round Ly6G^+^ cells.

### 2.2. T Cell Proliferation with S. aureus SAC-Exposed Murine CD11b^+^ Gr^+^ Bone Marrow Cells

**Figure 2:**
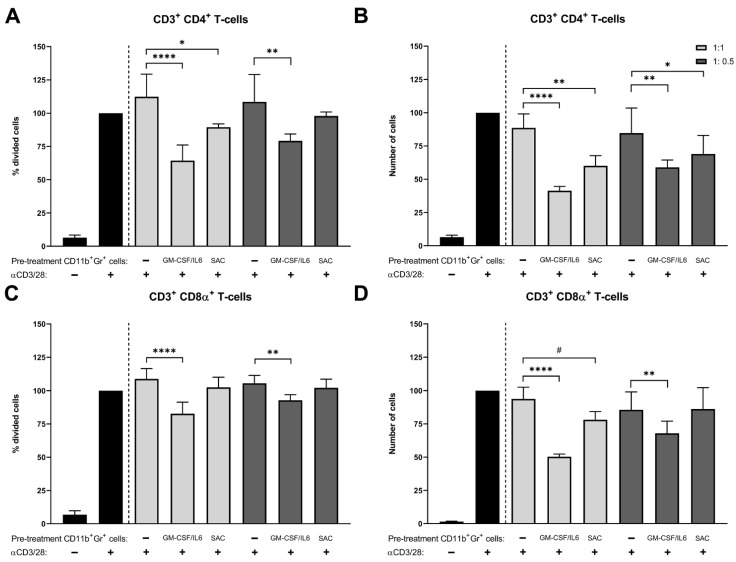
T cell proliferation assays with negative control, positive control, or *S. aureus* SAC-exposed murine monocytes and neutrophils. (**A**) Proliferation rates and (**B**) absolute cell numbers of CD3^+^ CD4^+^ T cells and (**C**) proliferation rates and (**D**) absolute cell numbers of CD3^+^ CD8α^+^ T cells were determined with flow analysis of PKH-stained splenocytes monocultures (black) without or with anti-CD3/CD28 stimulation and from PKH-stained and anti-CD3/CD28-stimulated splenocytes co-cultured with CD11b^+^ Gr-1^+^ monocytes and neutrophils from negative control, positive control, or *S. aureus* SAC co-culture samples in a 1:1 ratio (light grey) or 1:0.5 ratio (dark grey). Data are means (±SD) and are from four independent experiments with three replicated per test. Statistical test used: Holm–Sidak’s multiple comparison test; ^#^ *p* ≤ 0.1, * *p* < 0.05, ** *p* < 0.01, and **** *p* < 0.0001.

To test whether SAC-exposed bone marrow monocytes and neutrophils have immunosuppressive abilities, bone marrow cells positive for CD11b and Gr-1 (an antibody against both Ly6C and Ly6G, hence for both monocytes and neutrophils) were isolated. Furthermore, the isolated cells were used in a T cell proliferation assay, whereby PKH26-stained splenocytes (mostly T cells) received an anti-CD3/28 stimulus to induce T cell activation and expansion. Next, proliferation rates and absolute cell numbers of the two major T cell subtypes CD4^+^ T helper cells and cytotoxic CD8α^+^ T cells were quantified, as these should decrease in the presence of immunosuppressive cells (Figure 2). Compared to the negative control, CD11b^+^ Gr-1^+^ bone marrow cells from the positive control when cultured at a 1:1 or 0.5:1 ratio with splenocytes significantly reduced the percentage of proliferated CD4^+^ T cells (*p* < 0.0001 and *p* = 0.0012, respectively; Figure 2A), as well as the absolute CD4^+^ T cells numbers (*p* < 0.0001 and *p* = 0.0014, respectively; Figure 2B). SAC-exposed CD11b^+^ Gr-1^+^ bone marrow cells co-cultured at a 1:1 ratio with splenocytes also significantly reduced the percentage of proliferated CD4^+^ T cells (*p* = 0.036; Figure 2A) and the absolute CD4^+^ T cells numbers (*p* = 0.0022; Figure 2B) compared to the negative control, although to a lesser extent than the positive control. These effects were less pronounced in the 0.5:1 ratio culture with splenocytes (*p* = 0.37 and *p* = 0.046, respectively). In comparison to the negative control, the positive control CD11b^+^ Gr-1^+^ bone marrow cells significantly decreased the CD8α+ T cell proliferation rates. This was true for both the 1:1 and 0.5:1 ratio cultures (*p* < 0.0001 and *p* = 0.0057, respectively; Figure 2C). Furthermore, positive control bone marrow cells significantly decreased absolute CD8α+ T cell numbers in both the 1:1 and 0.5:1 ratio cultures (*p* < 0.0001 and *p* = 0.0026, respectively; Figure 2D) compared to the negative control. However, the SAC-exposed CD11b^+^ Gr-1^+^ bone marrow cells had 15.6% lower absolute CD8α^+^ T cell numbers than the negative control (*p* = 0.10; Figure 2D). These results showed that the positive control CD11b^+^ Gr-1^+^ bone marrow cells indeed had immunosuppressive abilities towards both CD4^+^ T cells and CD8α^+^ T cells and so were deemed to be MDSCs. Given that the SAC co-cultured CD11b^+^ Gr-1^+^ bone marrow cells had significant immunosuppressive activity towards CD4^+^ T cells, the SAC-exposed CD11b^+^ Gr-1^+^ bone marrow cells, therefore, can also be deemed to be MDSCs.

### 2.3. Expression of the MDSC-Related Enzymes iNOS and Arg-1 of S. aureus SAC-Exposed Murine Bone Marrow Cells

**Figure 3:**
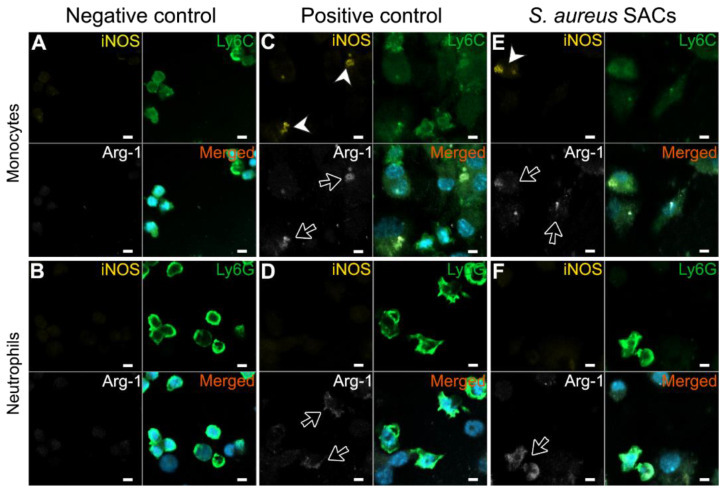
Representative immunofluorescent stains of monocytes or neutrophils for the MDSC-related enzymes iNOS and Arg-1. Untreated negative control (**A**) monocytes or (**B**) neutrophils, GM-CSF- and IL-6-treated positive control (**C**) monocytes, or (**D**) neutrophils of *S. aureus* SAC-exposed (**E**) monocytes or (**F**) neutrophils (green) were stained for iNOS (yellow; white arrowhead) and Arg-1 (white; black arrow) with DAPI as the nuclear stain (blue). Scale bar = 5 µm.

To explore whether the immunosuppressive abilities of SAC-exposed CD11b^+^ Gr-1^+^ bone marrow cells are possibly facilitated by the enzymes iNOS or Arg-1, immunofluorescent stains for iNOS and Arg-1 were performed. The monocytes and neutrophils from the negative control did not express iNOS or Arg-1 (Figure 3A,B, respectively). M-MDSCs from the positive control showed co-localization of the iNOS and Arg-1 stains at some spots (Figure 3C) and G-MDSCs from the positive control expressed Arg-1 (Figure 3D). Similar iNOS and Arg-1 expression patters were observed for the SAC-exposed murine bone marrow cells; the M-MDSCs were stained with both iNOS and Arg-1, while the G-MDSCs were only stained with Arg-1 (Figure 3E,F, respectively).

### 2.4. Secreted Proteins by the Murine Bone Marrow Cells When Exposed to In Vitro SACs

**Figure 4:**
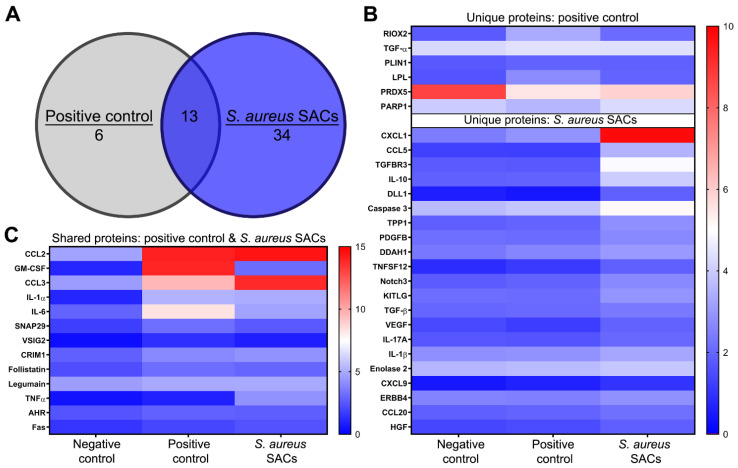
Protein biomarker analysis of supernatants from negative control, positive control, and *S. aureus* SAC-exposed murine bone marrow samples after 3 d culture. (**A**) Venn diagram highlighting the numbers of unique and shared significant proteins of positive control and SAC–bone marrow cultures in comparison to the negative control. Expression levels of (**B**) proteins that were significantly different in the positive control (upper part) or proteins of interest in the SAC–bone marrow cultures (lower part) compared to the negative control (0–5 = blue and 5–10 = red), as well as expression levels of (**C**) significant proteins in both the positive control and SAC–bone marrow cultures (0–7.5 = blue and 7.5–15 = red). Data are medians of normalized protein expression (NPX) values from three independent experiments, n = 9 per condition. Statistical test used: ANOVA with Tukey’s post hoc tests.

The secreted proteins from SAC-exposed murine bone marrow cells after 3 d culture were analyzed to possibly clarify which molecules might play a role in the immunosuppressive capabilities of these cells. The expression levels of a selection of secreted proteins indicative for various biological processes, including immune responses, chemotaxis, and cell proliferation from SAC-exposed and positive control bone marrow cells, were compared to the negative control (Supplementary Table S1, containing all data). In total, 53 proteins were significantly differently expressed as compared to the negative control, of which 34 were unique for SAC–bone marrow co-cultures, 6 were unique for positive control cultures, and 13 were shared for both SAC-exposed bone marrow and positive control cultures (Figure 4A). Proteins that were more expressed in positive control bone marrow cultures than the negative control were the enzymes RIOX1 and LPL, a modulator of lipid metabolism being PLIN1, and the cytokine TGF-α, whereas the enzymes PRDX5 and PARP1 were less expressed (Figure 4B, upper part). Proteins that were solely secreted at a significantly higher level in SAC–bone marrow co-cultures and were potentially MDSC-related were chemoattractants (for neutrophils, monocytes, Tregs, and Th17 cells); cytokines such as IL-10 and TGF-β; apoptosis or cell survival mediators; growth factors, including VEGF; and proteins of the Notch3, arginase/NO, glycolysis, or NF-κB signaling pathways (Figure 4B, lower part). Proteins secreted at a significantly higher level in both SAC–bone marrow and positive control cultures were chemokines (for neutrophils and monocytes), the growth factor GM-CSF, cytokines (IL-6, IL-1β, and TNFα), apoptosis or cell survival mediators, membrane proteins, the proangiogenic protein follistatin (FST), and the cysteine protease legumain (LGMN) (Figure 4C).

### 2.5. T Cell Proliferation Assays with Monocytes or Neutrophils from Non-Infected or S. aureus-Infected Mice

**Figure 5:**
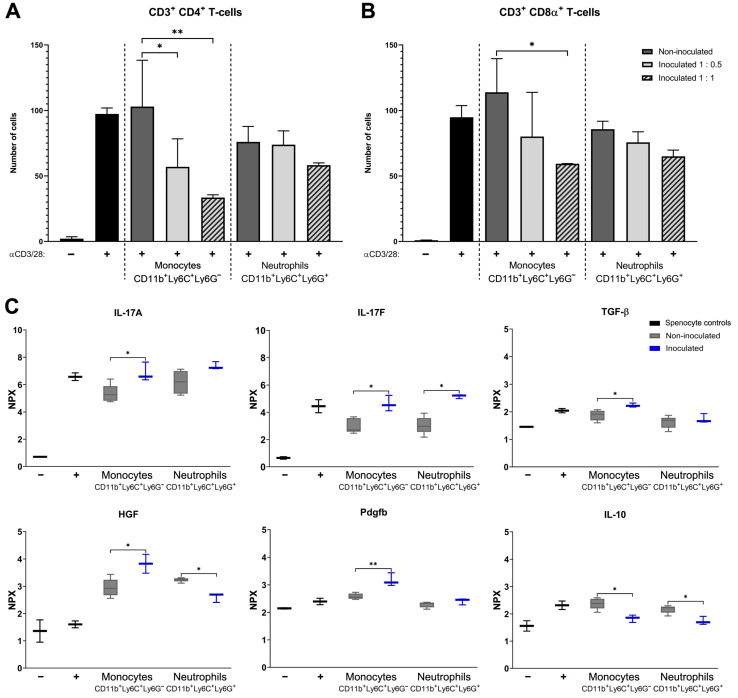
T cell proliferation assays with murine monocytes or neutrophils from non-inoculated or *S. aureus*-inoculated mice. (**A**) Absolute cell numbers of CD3^+^ CD4^+^ T cells and (**B**) absolute cell numbers of CD3^+^ CD8α^+^ T cells were determined with flow analysis of PKH-stained splenocytes monocultures (black) without or with anti-CD3/CD28 stimulation and from PKH-stained and anti-CD3/CD28-stimulated splenocytes co-cultured with CD11b^+^ Ly6C^+^ Ly6G^−^ monocytes or CD11b^+^ Ly6C^+^ Ly6G^+^ neutrophils from non-inoculated mice (1:1 ratio; dark-grey) or *S. aureus*-inoculated mice (1:0.5 ratio; light grey or 1:1 ratio; striped light grey). Results for T cell proliferation rates of these cultures were published previously [3]. Data are means (±SD) and n = 5. Statistical test used: Holm–Sidak’s multiple comparison test. (**C**) Normalized protein expression (NPX) values of proteins measured in supernatants of 4 d incubated PKH-stained splenocytes monocultures (black) without or with anti-CD3/CD28 stimulation and of PKH-stained and anti-CD3/CD28-stimulated splenocytes co-cultured 1:1 with CD11b^+^ Ly6C^+^ Ly6G^−^ monocytes or CD11b^+^ Ly6C^+^ Ly6G^+^ neutrophils from non-inoculated mice (grey) or *S. aureus*-inoculated mice (blue). Data are medians (±min/max) and n = 5. Statistical test used: ANOVA; * *p* < 0.05 and ** *p* < 0.01.

Monocytes or neutrophils isolated from bone marrow samples of non-infected mice or *S. aureus*-infected mice from a fracture fixation model were used in a T cell proliferation assay with PKH26-stained splenocytes to assess their effects on absolute CD4^+^ and CD8α^+^ T cell numbers. Compared to monocytes of non-infected mice, splenocyte co-cultures with monocytes from *S. aureus*-infected mice at 0.5:1 and 1:1 ratios had significant lower numbers of CD4^+^ T cells (*p* = 0.020 and *p* = 0.0092, respectively; Figure 5A) and were lower in numbers of CD8α^+^ T cells when co-cultured at a 1:1 ratio (*p* = 0.043; Figure 5B). Neither neutrophils from the *S. aureus*-infected mice or from the non-infected mice showed a difference in absolute T cell numbers. Co-cultures containing splenocytes and monocytes from *S. aureus*-infected mice and after a 4 d incubation showed significantly more IL-17A, IL-17F, transforming growth factor beta (TGF-β), hepatocyte growth factor (HGF), and platelet-derived growth factor-B (PDGFB) and significantly less IL-10 compared to co-cultures of splenocytes and monocytes from non-infected mice (Figure 5C).

### 2.6. Human Bone Marrow Cells Co-Cultured with S. aureus SACs

**Figure 6:**
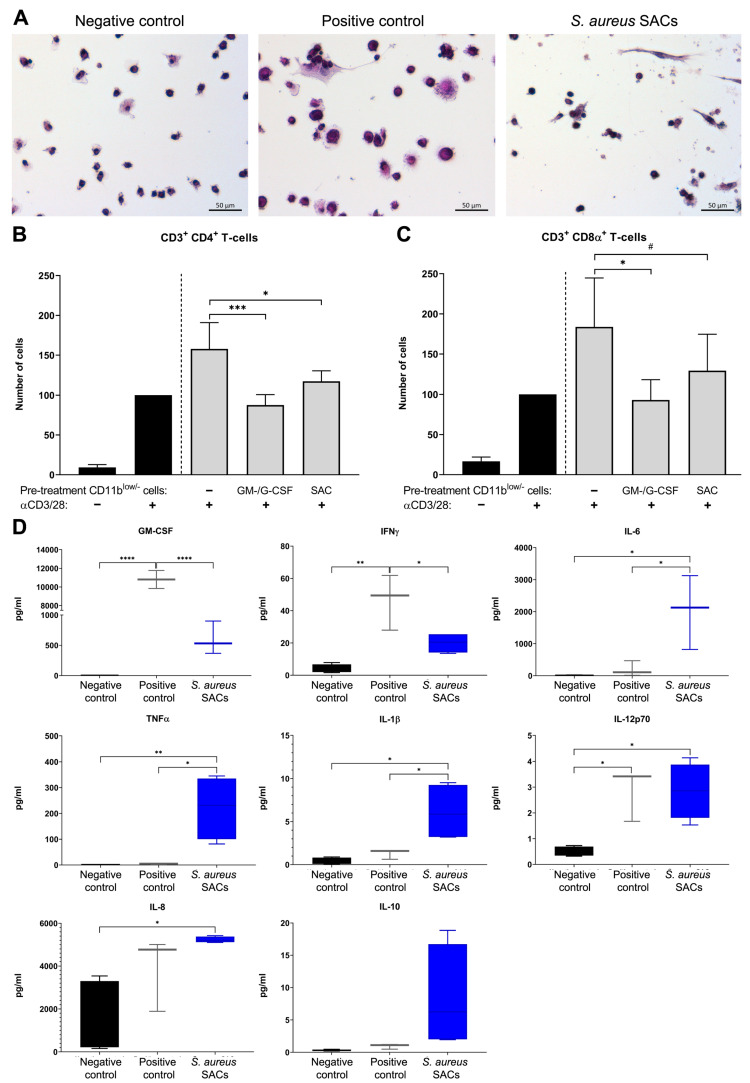
(**A**) Hematoxylin and eosin stains of negative control, positive control due to GM-CSF and G-CSF treatment, or *S. aureus* SAC-exposed human bone marrow cells. From the above-mentioned cultures, CD11b^low/−^ myeloid cells were isolated and used in a T cell proliferation assay with PKH-stained PBMC. Absolute cell numbers of (**B**) CD3^+^ CD4^+^ T cells and (**C**) of CD3^+^ CD8α^+^ T cells were determined with flow analysis of PKH-stained PBMC monocultures (black) without or with anti-CD3/CD28 stimulation and from PKH-stained and anti-CD3/CD28-stimulated PBMCs co-cultured with CD11b^low/−^ myeloid cells from negative control or *S. aureus* SAC co-culture samples in a 1:1 ratio (light grey). Data are means (±SD) and are from three (positive control) or four (negative control and S. aureus SACs) independent experiments with three or four different human bone marrow donors, respectively. (**D**) Analytes measured in 3-day-old culture supernatants of either negative control (black), positive control (grey), or SAC-exposed (blue) human bone marrow cell cultures present after 3 d culture. Data are medians (±min/max), n = 3 (positive control), n = 4 (negative control and *S. aureus* SACs). Statistical tests used: Holm–Sidak’s multiple comparison test; ^#^ *p* ≤ 0.1, * *p* < 0.05, ** *p* < 0.01 *** *p* < 0.001, and **** *p* < 0.0001.

CD33^+^ myeloid cells, which include monocytes and neutrophils, obtained from human bone marrow were co-cultured with in vitro SACs to examine whether human bone marrow cells develop an MDSC phenotype. CD33^+^ myeloid cells from human bone marrow were left untreated as the negative control or were treated with GM-CSF and G-CSF with 8% CO_2_ as the positive control. In the preparations of the negative control, only separate single cells with a round shape were observed, whereas in the positive control and SAC-exposed bone marrow cell preparations, in addition to clusters of cells, they also contained cells with an elongated appearance (Figure 6A).

Subsequently, CD11b^low/−^ myeloid cells, which were previously indicated to have the potential of becoming MDSCs [22,23,24], were isolated and used in a T cell proliferation assay with PKH26-stained human peripheral blood mononuclear cell (PBMCs; as a source of T cells) to assess their immunosuppressive abilities. Compared to CD11b^low/−^ myeloid cells from negative control samples, positive control and SAC-exposed CD11b^low/−^ myeloid cells significantly lowered the numbers of CD4^+^ T cells (*p* = 0.0002 and *p* = 0.010, respectively; Figure 6B). Furthermore, positive control CD11b^low/−^ myeloid cells significantly lowered CD8α^+^ T cell numbers compared to the negative control (*p* = 0.011; Figure 6B), whereas SAC-exposed CD11b^low/−^ myeloid cells lowered CD8α^+^ T cell numbers by 54.3% (*p* = 0.10; Figure 6C).

In cell culture supernatants of 3 d cultures, the positive control bone marrow cells GM-CSF, IFNγ, and IL-12p70 were found in abundance, whereas 3-day-old supernatants from SAC–bone marrow co-cultures contained elevated levels of GM-CSF, IL-6, TNFα, IL-1β, IL-12p70, IL-8, and IL-10 (Figure 6D).

## 3. Discussion

MDSCs have been associated with chronic *S. aureus* infections [3,13,14,15,16,17,18], and recently it has been suggested that cells close to SACs and within abscesses in *S. aureus* bone infections in mice might be MDSCs [3]. To investigate whether SACs cause MDSC expansion, we co-cultured human and murine bone marrow cells with our previously developed 3D in vitro SAC model and showed that indeed SAC-exposed human and murine bone marrow cells had immunosuppressive abilities and lowered absolute T cell numbers in a similar manner as monocytic MDSCs isolated from mice with an *S. aureus* bone infection. Furthermore, we characterized possible mediators that might be linked to MDSC expansion, activation, and immunosuppressive functioning due to SACs. This is, to the best of our knowledge, the first time that a direct link between SACs and MDSC induction has been reported.

As mentioned above, factors involved in MDSC expansion include GM-CSF, G-CSF, M-CSF, VEGF, and IL-6. These factors prevent immature myeloid cells from differentiating into mature cells by increasing STAT3 signaling [7,10,11,12]. Increased levels of IL-6 were detected in SAC–human and murine bone marrow cell co-cultures, which suggests that at least IL-6 could be involved in the expansion of MDSCs in these cultures. Previously, it has been shown that *S. aureus* biofilm-induced MDSCs have increased IL-6 expression compared to non-treated cells [17], in line with this study. GM-CSF and VEGF were also present in our SAC-exposed bone marrow cultures, suggesting that these factors also possibly play a role in MDSC expansion due to SACs in vitro.

Furthermore, TLR activation by *S. aureus* lipoproteins can result in MDSC expansion and can regulate MDSC functioning; during an *S. aureus* skin infection, resident skin cells were stimulated to secrete IL-6 by TLR2 activation, which resulted in recruitment and accumulation of MDSCs [13], while TLRs regulate the immunosuppressive function of MDSCs via the upregulation of iNOS and Arg-1 through MyD88 and NF-κB signaling [11]. In contrast, a lack of TLR activation has been reported to be beneficial for MDSC maintenance by suppressing maturation of the cells as well [25,26,27]. It is likely that TLR ligands of *S. aureus* that grow in SAC form are not easily accessible for immune cells, since SACs are covered with a fibrin pseudocapsule, which blocks access to immune cells [21], and possibly are not well released from the SAC. Therefore, the SACs might not provide or secrete the proper stimuli to activate TLRs, such as TLR9 [25,26,27], of the bone marrow myeloid cells in order for them to mature, forcing the cells to remain immature and facilitating the maintenance of the MDSCs. In line with this hypothesis, co-culturing of dispersed free *S. aureus* with murine bone marrow cells resulted in different monocyte and neutrophil ratios than co-culturing with in vitro SACs. Dispersed *S. aureus*-exposed monocytes and neutrophils had a different appearance than SAC-exposed monocytes and neutrophils.

Hypoxia is another driving factor of MDSC differentiation and functioning through HIF-α stabilization and transcription of HIF target genes [7,28]. The *S. aureus* SACs elaborate their fibrin network, other than the fibrin pseudocapsule, when in culture with bone marrow cells. It may be that given that the bone marrow cells are covered within this fibrin web, but possibly also because *S. aureus* uses oxygen, the growth conditions for the bone cells are more hypoxic and HIF-α stabilization with subsequent effects occurs, resulting in MDSC expansion from the bone marrow cells. During hypoxia the cells will use glycolysis as a metabolic pathway, a metabolic characteristic of MDSCs [7], and hypoxia can trigger autophagy [28]. Interestingly, the presence of proteins indicating that glycolysis and autophagy (Eno2, and Snap29 and TPP1, respectively) occurred in the SAC–bone marrow co-culture suggest that hypoxia might be a factor of MDSC induction due to *S. aureus* SACs. Future research clarifying this is required.

Prolonged exposure to pro-inflammatory cytokines leads to the activation of MDSCs [11,12]. Indeed, in the in vitro SAC–human and murine bone marrow cell co-cultures, elevated levels of the pro-inflammatory cytokines IL-1β and TNFα were found. Additionally, the cytokine IL-12p70 was found in high concentrations in SAC-exposed human bone marrow cultures. The cytokine IL-12 has been linked to MDSC recruitment in an *S. aureus* prosthetic joint infection in mice and was found to be an important factor in the persistent anti-inflammatory environment associated with biofilm [15].

Factors that contribute to the immunosuppressive activity of MDSCs are the enzymes Arg-1 (M-MDSCs and G-MDSCs) and iNOS (M-MDSCs) and their downstream metabolites, such as NO and ROS, as well as the cytokines IL-10 and TGF-β [8]. SAC-exposed monocytes indeed expressed Arg-1 and iNOS, while SAC-exposed neutrophils expressed Arg-1, indicating that their immunosuppressive actions might be mediated by these enzymes. Further research is required to quantify these or downstream metabolites such as NO, kynurenines, ornithine, urea, and polyamines [8]. Additionally, IL-10 and TGF-β were present in SAC–bone marrow cultures and might also contribute to the immunosuppressive abilities of SAC-exposed monocytes and neutrophils. In line with our finding, *S. aureus* biofilm-induced MDSCs showed increased expression of Arg-1, iNOS, and IL-10 [14,16,17] and cells from *S. aureus*-infected mice secreted high levels of IL-10 and TGF-β compared to cells of non-infected mice, although it has been argued that cell–cell proximity is more important for the immunosuppressive abilities of the *S. aureus*-exposed cells than IL-10 and TGF-β [18].

Aside from in vitro SACs causing MDSC expansion and activation in an indirect manner, by stimulating eukaryotic cells to secrete cytokines and growth factors, *S. aureus* might also directly induce MDSC expansion and activation. Recently, it has been reported that staphylococcal enterotoxins A and B cause G-MDSC expansion from human PBMCs, which were able to reduce T cell proliferation [29]. Furthermore, *S. aureus* biofilm, which was unable to secrete D- or L-lactate, lowered the secretion of IL-10 by MDSCs [30], while the synovial fluid of patients with prosthetic joint infection, which is known to contain MDSC-like cells [31], showed elevated levels of lactate [30]. Investigations into whether these above-mentioned *S. aureus* proteins are involved in MDSC expansion due to in vitro and in vivo SACs have yet to be performed and are the subject of current studies.

It appears that in vitro SACs triggered similar expansion and activation mechanisms in murine and human bone marrow cells. However, the immunosuppression of CD4^+^ and CD8α^+^ T cells by the SAC-exposed human CD11b^low/−^ myeloid cells was mainly through lowering their absolute numbers, whereas SAC-exposed murine CD11b^+^ Gr-1^+^ also decreased the proliferation rates of CD4^+^ T cells. The similarities between the in vitro and ex vivo cultures were the detected proteins HGF, PDGFB, IL-10, TGF-β, and IL-17A. The proteins HGF and PDGFB were both involved in fibrosis [32], which is especially interesting given that fibrosis is required to encapsulate the SAC and immune cells around SAC to form an abscess. Whether MDSCs might facilitate fibrosis, and subsequently whether in this manner they help *S. aureus* within SACs to persist, is important to investigate in the future.

In this study, the effects of SAC-exposed bone marrow monocytes and neutrophils on CD4^+^ and CD8 α^+^ T cells were investigated. However, it is known from cancer research that the tumor microenvironment, which MDSCs are part of, is immune-cell-rich and that other cells such as M2 macrophages, dendritic cells, and Tregs also facilitate the immunosuppressive milieu [10,33]. It would be interesting to examine the influence of SAC-induced MDSCs on these cell types, while Tregs and macrophages would also be especially be interesting candidates; both are induced by MDSCs through IL-10 and TGF-β [10,34], and these cytokines were present in SAC–bone marrow co-cultures. Additionally, MDSCs can suppress B cells, NK cells, dendritic cells, and macrophages [10,35], and little is known about how these cells are impacted by *S. aureus* (SAC)-induced MDSCs. This might also be studied in the future with the experimental setup used in the present study. MDSCs, as part of the tumor microenvironment, not only affect immune cells, but also stromal cells [36,37]. It would be interesting to clarify whether SAC-induced MDSCs also share this activity.

Besides the similarities in the tumor microenvironment and *S. aureus* abscesses, there are important differences. In *S. aureus* abscesses there is a viable core, the fibrin-encapsulated SAC [3], whereas in a tumor there is a necrotic core due to poor diffusion of oxygen and nutrients throughout the tumor [10,38,39]. The SAC’s fibrin pseudocapsule acts as a physical barrier for the immune cells [21], which is something that immune cells within a tumor do not encounter [38]. Furthermore, *S. aureus* within SAC structures may secrete a variety of virulence factors such as staphylococcal enterotoxins [40], staphylococcal protein A (SpA) [41,42], and pore-forming toxins [43] that actively and directly target not only innate immune cells, but also lymphoid cells.

We did not distinguish between M-MDSCs or G-MDSCs for the T cell proliferation assays with in vitro SAC-exposed bone marrow cells. It would be interesting to assess whether SAC-induced M-MDSCs and G-MDSCs possibly differ in their immunosuppressive actions, as was observed for the ex vivo cultures with monocytes or neutrophils from *S. aureus*-infected mice.

By using a 3D in vitro SAC model, we have demonstrated that immunosuppressive MDSCs, which mainly lowered T cell numbers, are induced from both murine and human bone marrow cells by the presence of *S. aureus* SACs. Furthermore, mediators in the expansion, activation, and immunosuppressive activity of the SAC-induced MDSCs were identified. Inhibition of these mediators, and thus of the expansion and activities of MDSCs, could possibly prevent establishment of the immunosuppressive environment associated with chronic, persistent *S. aureus* infections, and might provide an additional strategy for treating chronic *S. aureus* infections.

## 4. Materials and Methods

### 4.1. Bacteria

The clinical isolate *S. aureus* JAR 06.01.31 (the culture collection of Switzerland (CCOS) number 890, Wädenswill, Switzerland), obtained from a patient with an orthopaedic device-related infection [44], was used in this study. *S. epidermidis* O-47, originally isolated from a patient at the Institute Für Medizinische Mikrobiologie und Hygiene, University of Cologne (Germany) [45], was used as a coagulase-negative strain.

### 4.2. In Vitro SAC Model

In vitro SACs were produced as described previously [21] with some minor adjustments. Briefly, 10 µL collagen gel was prepared from rat collagen type I solution (1.78 mg/mL, pH 7.4; Gibco, Basel, Switzerland) following the manufacturer’s instructions and was added to a 24-well Transwell system (polyester membrane with a porosity of 0.4 µm; Corning Life Sciences B.V., Amsterdam, The Netherlands) and left to polymerize for 1 h at 37 °C in a humidified incubator. After 1 h, a 25 µL bacterial solution containing approximately 14 colony forming units (CFUs) of *S. aureus* JAR 06.01.31 was pipetted on top of the collagen gel together with 75 µL homogenized collagen and 300 µL pooled human plasma (Regional Blood Donation Service SRK Graubünden, Chur, Switzerland). Samples were incubated overnight at 37 °C and an additional 300 µL pooled human plasma was supplied after 5 h of incubation (Figure 1A).

In addition to *S. aureus*, the same procedure was applied to *S. epidermidis* O-47, resulting in small aggregates of the bacterium within the gel. As an additional control, approximately 2.5 × 10^5^ log-phase *S. aureus* JAR 06.01.31 were added to 100 µL collagen gel and mixed to obtain a dispersed bacterial presence throughout the gel, but no concentrated, fibrin-encased SAC.

### 4.3. Murine Bone Marrow Cell and Splenocyte Isolation

After washing the femoral and tibial bones in Hank’s buffered salt solution (HBSS; Gibco) and removing the outer ends of the bones, the bone marrow cells were flushed out with a 26G × 1” needle attached to a 2 mL syringe (both Braunn, Davos Platz, Switzerland) containing HBSS. The collected cells were passed through a 70 µm cell strainer, centrifuged at 300× *g* for 6 min at 4 °C, the supernatant was removed, and the cell pellet was incubated in 5 mL erythrocyte lysis buffer (15 mM NH_4_Cl, 1 µM KHCO_3_ and 10 µM disodium ethylenediaminetetraacetic acid; all Sigma-Aldrich, Buchs, Switzerland) for 5 min at RT. Subsequently, 25 mL Roswell Park Memorial Institute 1640 (RPMI; Gibco) supplemented with 3% fetal bovine serum (FBS; Sigma-Aldrich) was added to neutralize the lysis buffer. The washed and isolated cells were centrifuged at 300× *g* for 6 min at 4 °C, resuspended in freezing medium consisting of RPMI with 40% FBS and 10% dimethyl sulfoxide (DMSO; Sigma-Aldrich), and stored in liquid nitrogen until further use.

Spleens were pushed through a 70 µm cell strainer that was placed on a 50 mL tube with the plunger of a 5 mL syringe to isolate splenocytes. The cell strainer was washed with 5 mL RPMI with 3% FBS, the cell suspension was centrifuged at 300× *g* for 6 min at 4 °C, the supernatant was discarded, and the lysis buffer was applied for 4 min at RT. After adding 25 mL RPMI with 3% FBS, cells were spun down at 300× *g* for 6 min at 4 °C, washed with 10 mL RPMI with 10% FBS, centrifuged at 300× *g* for 6 min at 4 °C, and resuspended in 20 mL RPMI with 10% FBS. The splenocyte suspension was filtered with a 40 µm cell strainer, resuspended in freezing medium, and stored in liquid nitrogen until further use.

### 4.4. In Vitro SAC–Murine Bone Marrow Co-Cultures

After overnight culture, two in vitro SAC samples were removed from the 24-well Transwell plate and placed into a 6-well plate. Murine bone marrow cells (1 × 10^6^ per well) were cultured in RPMI with 10% FBS, 1 mM sodium pyruvate (Sigma-Aldrich), and 50 µM β-mercaptoethanol (Roth AG, Arlesheim, Switzerland), and either did not receive any further supplements (negative control), were supplemented with 40 ng/mL IL-6 and GM-CSF (positive control; both Peprotech, London, UK), or were exposed to in vitro SACs, then all three conditions were cultured for 3 d at 37 °C with 5% CO_2_. Thereafter, in vitro SACs were removed from the wells and discarded and the bone marrow cells from the three different conditions were collected on ice using a cell scraper. Approximately 50,000 cells were kept aside; stained for CD11b-APC, Ly6G-FITC, Ly6C-PE (all Biolegend, Fell, Germany), and DAPI (Sigma-Aldrich); and analyzed with the BD FACSDiva flow cytometer (BD Bioscience, Allschwil, Switzerland). From the remaining bone marrow cells, CD11b^+^ Gr^+^ cells were purified using the EasySep™ Mouse MDSC (CD11b^+^ Gr-1^+^) isolation kit (StemCell, Saint Egrève, France) following the manufacturer’s protocol.

For the co-cultures with *S. epidermidis* aggregates or *S. aureus* in gel, 2.5 × 10^5^ murine bone marrow cells were added in RPMI supplemented with 10% FBS, 1 mM sodium pyruvate, and 50 µM β-mercaptoethanol to a 24-well plate and cultured for 3 d at 37 °C with 5% CO_2_. Then, the *S. epidermidis* aggregates or *S. aureus* in gel were removed from the samples; the bone marrow cells were collected on ice by using a cell scraper; stained for CD11b-APC, Ly6G-FITC, Ly6C-PE, and DAPI; and acquired with the BD FACSDiva flow cytometer.

### 4.5. Murine T Cell Proliferation Assay

Splenocytes were stained with the PKH26 membrane dye (Sigma-Aldrich) following the manufacturer’s instructions. For the T cell proliferation assay, the PKH26-stained splenocytes were plated at 2.5 × 10^4^/well on a 96-well plate in RPMI supplemented with 10% FBS, 1% (*v*/*v*) penicillin/streptomycin solution (Gibco), 1 mM sodium pyruvate, and 50 µM β-mercaptoethanol. Splenocytes were either unstimulated and cultured as a monoculture or splenocytes were stimulated with murine CD3/CD28 Dynabeads (Thermofisher Scientific, Darmstadt, Germany; 1:1 ratio) and 30 U/mL murine rIL-2 (Peprotech) and cultured as a monoculture or as a co-culture with purified CD11b^+^ Gr^+^ bone marrow cells in a 1:1 or 1:0.5 cell ratio. The cells were incubated for 3 d at 37 °C with 5% CO_2_, then subsequently the T cells were assessed for their proliferation rate and cell number by flow cytometry. Cells were stained for CD3-FITC, CD4-Alexa Fluor 700, CD8α-APC antibodies (all Biolegend), and DAPI and collected in TruCount tubes (BD Bioscience). Proliferation was normalized to T cells stimulated with CD3/CD28 Dynabeads and rIL-2, which were set to 100%.

### 4.6. Human Bone Marrow Cell and PBMC Isolation

CD33^+^ myeloid cells were enriched from human bone marrow aspirates of four different donors using the RosetteSep™ HLA myeloid cell enrichment kit (StemCell). In short, bone marrow aspirates were incubated with RosetteSep™ HLA cocktail for 20 min at RT, diluted 1:1 with HBSS with 2% FBS, layered on top of 10 mL RosetteSep™ DM-M Density Medium, and centrifuged at 330× *g* for 25 min without brakes. The enriched cells were removed and the remaining erythrocytes were lysed using 5 mL lysis buffer as mentioned above.

PBMCs were isolated from whole blood by performing density gradient centrifugation with Lymphoprep (StemCell) as the density gradient medium and by following the manufacturer’s protocols.

### 4.7. In Vitro SAC–Human Bone Marrow Co-Cultures

Myeloid enriched bone marrow cells were cultured 2 × 10^6^ per well of a 6-well plate in Iscove’s Modified Dulbecco’s Medium (IMDM; Avantor, Dietikon, Switzerland) supplemented with 10% FBS, 0.01 M HEPES (Thermofisher Scientific), 0.55 mM arginine (Sigma-Aldrich), 0.24 mM asparagine (Sigma-Aldrich), 1.5 mM glutamine (Sigma-Aldrich), and 50 µM β-mercaptoethanol for 3 d at 37 °C with 5% CO_2_ [24]. Some of the cells were also exposed to overnight-grown in vitro SACs or were supplemented with 40 ng/mL GM-CSF and G-CSF (Peprotech) and cultured with 8% CO_2_ [24]. Cells were harvested as mentioned above and CD11b^low/−^ cells were isolated with the EasySep™ “Do-It-Yourself” positive selection kit using an anti-human CD11b antibody (both StemCell) as the selection antibody. The kit was used as described by the manufacturer, but instead of collecting the isolated CD11b^+^ cells, the remaining CD11b^low/−^ cells were collected.

### 4.8. Human T Cell Proliferation Assay

The CD11b^low/−^ cells were co-cultured with 2.5 × 10^4^ PKH26-stained PBMCs at a 1:1 ratio in RPMI 1640 medium for SILAC (Thermofisher Scientific) with 150 µM arginine, 218.5 µM L-lysine monohydrochloride (Sigma-Aldrich), human CD3/CD28 Dynabeads (Thermofisher Scientific; 1:1 ratio), and 30 U/mL human rIL-2 (Peprotech) for 4 d at 37 °C with 5% CO_2_. PBMCs were also cultured either unstimulated or stimulated with human CD3/CD28 Dynabeads and 30 U/mL human rIL-2. Cells were collected; stained for CD3-BV605, CD4-FITC, CD8α-APC antibodies (all Biolegend), and DAPI; transferred to TruCount tubes; then T cells numbers were assessed with flow analysis. The number of T cells from the stimulated PBMC monocultures was set to 100% and used for normalization of the other samples.

### 4.9. In Vivo Samples

Ex vivo cell culture supernatant from splenocytes co-cultured with either FACS-sorted CD11b^+^ Ly6C^+^ Ly6G^−^ or CD11b^+^ Ly6C^+^ Ly6G^+^ bone marrow cells from infected or non-infected mice were obtained from a study approved by the ethical committee of the canton of Graubünden in Switzerland (approval number 2019_10), which was previously reported [3]. In short, specific pathogen-free (SPF) C57Bl/6N female mice (Charles River, Sulzland, Germany) aged 20 to 28 weeks received as surgical intervention a double osteotomy of the left femur after the bone was stabilized with a titanium 6-hole MouseFix locking plate (RISystems AG, Davos Platz, Switzerland) by inserting the 4 outermost screws. The 2 mm segment that was created was taken out and inoculated with 1 µL PBS containing approximately 1 × 10^4^ CFU of *S. aureus* JAR 06.01.31 or 1 µL sterile PBS. The inoculum or saline was allowed to absorb into the bone for 3 min and the segment was placed back into its original place without fixation. The fascia lata and the skin were closed with continuous sutures (5-0 Vicryl rapide, Ethicon, Courcelles, Belgium). At 21 days post-operative, animals were sacrificed and bone marrow for the left femurs was isolated as mentioned above. Monocytes (CD11b^+^ Ly6C^+^ Ly6G^−^) or neutrophils (CD11b^+^ Ly6C^+^ Ly6G^+^) from the isolated bone marrow were sorted with a BD FACSDiva instrument (BD Bioscience) using anti-CD11b-APC, anti Ly6G-FITC, anti Ly6C-PE (all Biolegend), and DAPI. Cells were sorted with an efficiency rate above 86% and purified cells were kept on ice until further use. For the FACS-sorted monocytes or neutrophils, a murine T cell proliferation assay was performed.

### 4.10. Immunofluorescent and Histochemical Stains

For immunofluorescent stains, fixed samples were first blocked with 1:20-diluted animal-free blocker (Vector Laboratories, Burlingame, CA, USA) in PBS with 1% Triton X (PBS-T; Sigma-Aldrich) for 1 h at RT. Blocking buffer was removed and primary antibodies (Table 1) diluted to 1:200 were added to the samples and incubated for 1 h at RT. Subsequently, samples were washed 3 times with PBS-T for 5 min, then if applicable secondary antibodies (1:200 dilution; Table 1) were added to the samples and incubated for 30 min in the dark at RT. Lastly, samples were washed 3 times with PBS-T for 5 min and a PBS solution with DAPI was added.

Hematoxylin and eosin stains were performed with Mayer’s hematoxylin and a 0.25% eosin solution (both Sigma-Aldrich).

### 4.11. Protein Biomarker Analysis

In vitro cell culture supernatants of murine bone marrow cells (negative control, positive control, or co-cultures with in vitro SAC) and ex vivo cell culture supernatant from splenocytes co-cultured with either FACS sorted CD11b^+^ Ly6C^+^ Ly6G^−^ or CD11b^+^ Ly6C^+^ Ly6G^+^ bone marrow cells from infected or non-infected mice were analyzed with the mouse exploratory panel (Olink, Uppsala, Sweden) following the manufacturer’s instructions. Data are shown as normalized protein expression values; the protein expression value is an arbitrary unit in a Log2 scale of Olink calculated from Ct values.

### 4.12. Cytokine and Growth Factor Measurements

In vitro cell culture supernatants of human bone marrow cells (negative control, positive control, or co-cultures with in vitro SAC) were assessed for the presence of GM-CSF, IFN-γ, IL-6, TNFα, IL-1β, IL-12p70, IL-8, and IL-10 using U-plex multiplex assays (MSD, Rockville, MD, USA).

### 4.13. Data Analysis

Flow cytometric data were analyzed with Kaluza Analysis Software (Beckman Coulter Life Sciences, Indianapolis, IN, USA). Statistical analysis of the Olink data was performed used the online Olink Insights Stat Analysis tool (Olink) using ANOVA with Tukey’s post hoc test as the statistical test, while Olink data visualization was performed with GraphPad Prism 8 (GraphPad Software, San Diego, CA, USA). Statistical analysis of the flow cytometry data was performed with GraphPad Prism 8. The normality of the data was checked with a Shapiro–Wilk test, and subsequently the data were analyzed with Holm–Sidak or Tukey’s multiple comparison test. Here, *p*-values of <0.05 were considered statistically significant.

## Figures and Tables

**Table 1:** A list of all primary antibodies used and their accompanying secondary antibodies.

Primary Antibody	Dilution	Secondary Antibody
Rat monoclonal anti-Ly6G antibody (BD Bioscience, 551459) conjugated with Alexa Fluor^®^ 647 (Abcam, ab269823)	1:50	-
Rat monoclonal anti-Ly-6C antibody (Biolegend, 128002) conjugated with Alexa Fluor^®^ 488 (Abcam, ab236553)	1:25	-
Rat monoclonal anti-Ly6G antibody	1:50	Goat anti-rat IgG Alexa Fluor 488
Rat monoclonal anti-Ly-6C antibody	1:25	Goat anti-rat IgG Alexa Fluor 488
Rabbit polyclonal anti-iNOS antibody (Abcam, ab15323)	1:50	Goat anti-rabbit IgG Alexa Fluor 568
Goat polyclonal anti-arginase 1 antibody (Genetex, GTX88484)	1:200	Donkey anti-goat IgG Alexa Fluor Plus 647

## Data Availability

The data presented in this study are available on request from the corresponding author.

## Supplementary Materials

The following are available online at https://www.mdpi.com/article/10.3390/pathogens10111446/s1, Table S1: Protein biomarker analysis of murine positive control and SAC-exposed bone marrow cultures in comparison to negative control bone marrow cells.
