# Supplementary material for: In Vitro 3D Staphylococcus aureus Abscess Communities Induce Bone Marrow Cells to Expand into Myeloid-Derived Suppressor Cells
Source: Pathogens. 2021 Nov 6;10(11):1446. doi: 10.3390/pathogens10111446 (PMC8622274; doi:10.3390/pathogens10111446)
Supplement: Supplementary file 1 [file pathogens-10-01446-s001.zip › pathogens-1424464-supplementary.pdf]

Table S1. Proteomics of murine positive control and SAC-exposed bone marrow cultures in comparison to negative control bone marrow cells. Significantly differently expressed proteins are in bold.

	Positive control				*S. aureus* SACs			
**ANALYTE**	NPX	Min	Max	p-value	NPX	Min	Max	p-value
**ACVRL1**	0.080198	-0.10633	0.266722	0.53909	0.506429	0.319904	0.692953	**1.51E-06**
**ADAM23**	-0.01126	-0.33606	0.313531	0.995875	0.057263	-0.26753	0.382057	0.899103
**AHR**	0.519393	0.118256	0.920531	**0.009555**	0.420159	0.019021	0.821296	**0.038837**
**APBB1IP**	-0.0693	-0.34946	0.210868	0.811916	0.034648	-0.24552	0.314813	0.948893
**AXIN1**	0.093668	-0.14632	0.333658	0.599518	0.106194	-0.1338	0.346185	0.520293
**CA13**	0.188117	-0.22917	0.605408	0.507943	0.138142	-0.27915	0.555433	0.690413
**CANT1**	-0.0036	-0.32141	0.314206	0.999559	0.31755	-0.00026	0.635356	0.050212
**CASP3**	0.162021	-0.34083	0.664871	0.703805	1.607817	1.104966	2.110667	**9.60E-08**
**CCL2**	8.81005	8.017551	9.602549	**2.10E-14**	9.148914	8.356415	9.941414	**2.10E-14**
**CCL20**	0.026476	-0.31521	0.368161	0.979587	0.36247	0.020785	0.704155	**0.03612**
**CCL3**	5.38239	4.80773	5.95705	**2.11E-14**	9.413543	8.838884	9.988203	**2.10E-14**
**CCL5**	-0.05182	-0.40103	0.297393	0.927331	2.184746	1.835534	2.533957	**1.55E-13**
**CDH6**	-0.00492	-0.1965	0.186668	0.997737	0.049103	-0.14248	0.240689	0.799667
**CLMP**	0.10242	-0.24738	0.452218	0.747615	0.04704	-0.30276	0.396838	0.93988
**CLSTN2**	0.083914	-0.26573	0.433555	0.821784	0.205938	-0.1437	0.555578	0.322245
**CNTN1**	-0.00673	-0.52663	0.513157	0.999423	2.099522	1.579631	2.619413	**1.23E-09**
**CNTN4**	-0.0538	-0.58783	0.480234	0.965765	2.014092	1.480063	2.548122	**4.61E-09**
**CPE**	0.117222	-0.12702	0.361461	0.465457	0.124439	-0.1198	0.368677	0.423963
**CRIM1**	0.904332	0.541812	1.266853	**5.66E-06**	1.319222	0.956702	1.681743	**9.08E-09**
**CSF2**	12.41882	11.65896	13.17868	**2.10E-14**	1.680909	0.921049	2.440769	**3.21E-05**
**CXCL1**	0.495879	-0.38927	1.381028	0.357239	8.059759	7.17461	8.944908	**2.11E-14**
**CXCL9**	0.161654	-0.36769	0.690995	0.72902	0.587522	0.058182	1.116863	**0.027592**
**CYR61**	0.174816	-0.17844	0.528071	0.444285	0.385834	0.032579	0.73909	0.051
**DCTN2**	-0.14064	-0.49816	0.216891	0.59482	0.088564	-0.26896	0.446092	0.811418
**DDAH1**	0.2086	-0.04211	0.459306	0.115792	0.668212	0.417506	0.918918	**2.03E-06**
**DLK1**	0.025326	-0.56809	0.618744	0.993759	2.261857	1.668439	2.855275	**3.77E-09**
**DLL1**	-0.05614	-0.44964	0.337354	0.93261	1.280693	0.887197	1.67419	**7.01E-08**
**EDA2R**	0.134764	-0.05114	0.320672	0.187645	0.163271	-0.02264	0.349179	0.092779
**ENO2**	0.102199	-0.16202	0.36642	0.604902	0.312828	0.048607	0.577049	**0.018189**
**EPCAM**	0.086451	-0.4128	0.585706	0.902476	2.609799	2.110544	3.109054	**6.37E-12**
**EPO**	0.031233	-0.12164	0.184105	0.867108	0.054049	-0.09882	0.20692	0.656011
**ERBB4**	-0.06824	-0.33292	0.196436	0.79755	0.285387	0.020708	0.550065	**0.032852**
**FAS**	0.61854	0.093644	1.143436	**0.018774**	0.806113	0.281217	1.33101	**0.002221**
**FLI1**	-0.00022	-0.29004	0.289609	0.999998	0.151759	-0.13807	0.441583	0.404773
**FLRT2**	0.05412	-0.37678	0.485023	0.947334	0.131029	-0.29987	0.561932	0.730964
**FOXO1**	-0.00503	-0.27401	0.263956	0.998801	0.09932	-0.16966	0.368301	0.631898
**FST**	0.815567	0.438595	1.192539	**4.34E-05**	0.587228	0.210256	0.9642	**0.001938**
**FSTL3**	-0.02648	-0.2431	0.190139	0.950036	0.579132	0.362515	0.79575	**1.93E-06**
**GCG**	0.03534	-0.2574	0.328082	0.951235	0.016187	-0.27656	0.308929	0.989548

**GDNF**	0.170066	-0.13813	0.478262	0.367873	0.108646	-0.19955	0.416842	0.657604
**GFRA1**	-0.02679	-0.31688	0.263294	0.97113	0.413452	0.123364	0.703541	**0.004371**
**GHRL**	0.0107	-0.23806	0.259458	0.99366	0.164297	-0.08446	0.413054	0.245085
**HGF**	0.196818	-0.08861	0.482241	0.21774	0.365352	0.079929	0.650776	**0.010423**
**IGSF3**	-0.06644	-0.33758	0.204706	0.815058	-0.02151	-0.29266	0.249628	0.978608
**IL10**	-0.06541	-0.49268	0.36185	0.922842	2.157867	1.730602	2.585131	**1.32E-11**
**IL17A**	-0.01264	-0.39992	0.374638	0.996344	0.573626	0.186346	0.960905	**0.003108**
**IL17F**	-0.02632	-0.27593	0.223292	0.962557	0.20612	-0.04349	0.455732	0.119267
**IL1A**	4.194051	3.4792	4.908902	**5.53E-13**	4.300574	3.585724	5.015425	**3.31E-13**
**IL1B**	0.065698	-0.25462	0.386018	0.866158	0.443	0.122679	0.763321	**0.005646**
**IL23R**	-0.04612	-0.36224	0.270005	0.929666	0.142314	-0.17381	0.458436	0.508855
**IL5**	-0.15824	-0.4759	0.159417	0.439694	-0.14442	-0.46208	0.173235	0.502263
**IL6**	4.927272	3.871304	5.98324	**6.80E-11**	1.640776	0.584807	2.696744	**0.001986**
**ITGB1BP2**	0.076324	-0.19914	0.351791	0.770417	0.018151	-0.25732	0.293617	0.985192
**ITGB6**	-0.13787	-0.44078	0.165038	0.501518	0.082794	-0.22011	0.385701	0.775762
**KITLG**	0.014881	-0.3258	0.355562	0.993463	0.816732	0.476052	1.157413	**1.02E-05**
**LGMN**	0.353468	0.177983	0.528953	**0.000111**	0.309749	0.134264	0.485234	**0.000531**
**LPL**	1.049864	0.825866	1.273862	**6.20E-11**	0.209558	-0.01444	0.433556	0.069765
**MAP2K6**	0.133862	-0.06013	0.327856	0.217319	-0.02418	-0.21818	0.169811	0.948094
**MATN2**	0.221934	-0.29886	0.74273	0.544843	2.661233	2.140438	3.182029	**1.03E-11**
**MIA**	-0.19875	-0.70371	0.306221	0.594476	0.504191	-0.00078	1.009159	0.050406
**NADK**	0.147844	-0.42201	0.717694	0.79531	0.851318	0.281468	1.421168	**0.002873**
**NOTCH3**	0.089583	-0.24539	0.424555	0.784122	0.826023	0.491052	1.160995	**6.73E-06**
**NTF3**	0.025131	-0.2445	0.294765	0.970612	0.155987	-0.11365	0.425621	0.334743
**PAK4**	0.092524	-0.20802	0.393066	0.72534	0.336746	0.036204	0.637287	**0.026021**
**PARP1**	-0.58837	-1.09156	-0.08519	**0.019772**	0.047144	-0.45604	0.550328	0.970309
**PDGFB**	0.037697	-0.15819	0.233584	0.881089	0.525714	0.329827	0.721602	**1.82E-06**
**PLA2G4A**	0.348788	-0.00185	0.699426	0.051406	0.155657	-0.19498	0.506295	0.518162
**PLIN1**	0.34824	0.068069	0.628411	**0.012947**	0.224896	-0.05528	0.505066	0.132754
**PLXNA4**	0.008657	-0.34264	0.359954	0.997915	0.210167	-0.14113	0.561464	0.311453
**PPP1R2**	0.165978	-0.27827	0.610226	0.625172	0.334666	-0.10958	0.778913	0.165831
**PRDX5**	-2.78212	-4.5173	-1.04694	**0.00146**	-1.60032	-3.33551	0.134857	0.074592
**QDPR**	0.04046	-0.23112	0.312042	0.926772	0.15402	-0.11756	0.425602	0.348649
**RGMA**	-0.09465	-0.57274	0.383433	0.874636	1.706359	1.228274	2.184444	**1.30E-08**
**RIOX2**	1.541017	1.083306	1.998727	**3.81E-08**	0.397518	-0.06019	0.855228	0.097281
**S100A4**	0.341681	-0.15354	0.836906	0.217391	-0.15743	-0.65265	0.337795	0.710301
**SEZ6L2**	0.032767	-0.12533	0.190868	0.863556	0.055233	-0.10287	0.213335	0.66246
**SNAP29**	1.545736	1.041776	2.049695	**1.98E-07**	0.750219	0.246259	1.254178	**0.002968**
**TGFA**	0.196557	0.042878	0.350235	**0.010486**	0.12057	-0.03311	0.274249	0.144228
**TGFB1**	0.188951	-0.03432	0.412218	0.108228	0.50146	0.278194	0.724726	**2.60E-05**
**TGFBR3**	-0.05662	-0.60453	0.491283	0.964004	3.022201	2.474295	3.570107	**2.03E-12**
**TNF**	0.58382	0.203859	0.963781	**0.00221**	3.800423	3.420462	4.180385	**2.10E-14**
**TNFRSF11B**	-0.02754	-0.37662	0.321545	0.978854	0.788291	0.439211	1.137371	**2.41E-05**
**TNFRSF12A**	-0.01848	-0.28914	0.252186	0.984115	0.029839	-0.24082	0.300502	0.959149
**TNFSF12**	0.232146	-0.16932	0.633615	0.335071	1.004083	0.602614	1.405552	**5.44E-06**

**TNNI3**	0.40275	-0.14408	0.949576	0.178473	0.30913	-0.2377	0.855956	0.350884
**TNR**	0.053999	-0.21613	0.324123	0.872368	0.142816	-0.12731	0.41294	0.397983
**TPP1**	0.102314	-0.25834	0.462969	0.760883	1.05846	0.697806	1.419114	**4.21E-07**
**VEGFD**	-0.12666	-0.43677	0.183461	0.571776	0.5082	0.198084	0.818316	**0.001171**
**VSIG2**	0.837719	0.557565	1.117873	**3.83E-07**	0.483447	0.211657	0.755236	**0.000513**
**WFIKKN2**	0.156774	-0.08696	0.400506	0.2624	0.463532	0.219801	0.707263	**0.000224**
**WISP1**	-0.0803	-0.44002	0.279424	0.843672	0.229236	-0.13049	0.588957	0.268587
**YES1**	-0.06831	-0.37288	0.236261	0.842321	0.003289	-0.30128	0.307858	0.999599
